# 2,4-Dichlorophenoxyacetic acid degradation in methanogenic mixed cultures obtained from Brazilian Amazonian soil samples

**DOI:** 10.1007/s10532-021-09940-3

**Published:** 2021-04-20

**Authors:** Gunther Brucha, Andrea Aldas-Vargas, Zacchariah Ross, Peng Peng, Siavash Atashgahi, Hauke Smidt, Alette Langenhoff, Nora B. Sutton

**Affiliations:** 1 Environmental Technology, Wageningen University & Research, PO BOX 17, 6700 EV Wageningen, The Netherlands; 2 Institute of Science and Technology, Universidade Federal de Alfenas, Alfenas, Brazil; 3 Laboratory of Microbiology, Wageningen University & Research, Wageningen, The Netherlands

**Keywords:** Pesticide, 2,4-Dichlorophenoxyacetic acid, Methanogenic, Biodegradation, Soil, Microbial diversity

## Abstract

2,4-Dichlorophenoxyacetic acid (2,4-D) is the third most applied pesticide in Brazil to control broadleaf weeds in crop cultivation and pastures. Due to 2,4-D’s high mobility and long half-life under anoxic conditions, this herbicide has high probability for groundwater contamination. Bioremediation is an attractive solution for 2,4-D contaminated anoxic environments, but there is limited understanding of anaerobic 2,4-D biodegradation. In this study, methanogenic enrichment cultures were obtained from Amazonian top soil (0—40 cm) and deep soil (50 -80 cm below ground) that biotransform 2,4-D (5 µM) to 4-chlorophenol and phenol. When these cultures were transferred (10% v/v) to fresh medium containing 40 µM or 160 µM 2,4-D, the rate of 2,4-D degradation decreased, and biotransformation did not proceed beyond 4-chlorophenol and 2,4-dichlorophenol in the top and deep soil cultures, respectively. 16S rRNA gene sequencing and qPCR of a selection of microbes revealed no significant enrichment of known organohalide-respiring bacteria. Furthermore, a member of the genus *Cryptanaerobacter* was identified as possibly responsible for phenol conversion to benzoate in the top soil inoculated culture. Overall, these results demonstrate the effect of 2,4-D concentration on biodegradation and microbial community composition, which are both important factors when developing pesticide bioremediation technologies.

**Supplementary Information:**

The online version contains supplementary material available at 10.1007/s10532-021-09940-3.

## Introduction

The Brazilian agriculture sector has undergone enormous growth in recent decades, with the country now becoming one of the world’s largest supplier of soy, coffee, sugar cane and corn, among other commodities (FAO 2015). However, this rapid growth has relied heavily on the use of pesticides. In 2008, Brazil became the world’s largest market for pesticide import, and in 2015, 899 million litres of pesticides were used (Piganati et al. 2017). Pesticide use is regulated under a Federal law in Brazil since 1989 (ANVISA 2011), however, crop and freshwater monitoring has shown pesticide concentrations regularly exceed allowed levels and water quality criteria (Jardim and Caldas 2012; Albuquerque et al. 2016; Barbosa et al. 2015).

2,4-Dichlorophenoxyacetic acid (2,4-D), an auxin hormone mimic, is the third most widely applied pesticide in Brazil (Barbosa et al. 2015), used to control broadleaf weeds in cereal and sugarcane cultivation as well as pastures. For systemic hormonal action, 2,4-D is classified toxicologically in Brazil as class I (highly toxic) (ANVISA 2016) and as class III for environmental hazards (product dangerous to the environment) (Rebelo et al. 2010). Although 2,4-D was reported to have a relatively short aerobic soil half-life of 6.2 days (EPA 2005), its degradation rate is strongly influenced by several factors such as pH, temperature and moisture (Sandmann et al. 1988). Combined with 2,4-D’s high terrestrial mobility (EPA 2005), this enhances the potential for percolation through the soil and into groundwater systems. Once present in anoxic environments, including saturated agricultural land and aquifers, 2,4-D is considered ‘persistent’ to ‘highly persistent’ with a half-life of 41–333 days (EPA 2005). Therefore, the stability and mobility of 2,4-D within anoxic environmental systems causes concern for the transport to and contamination of offsite locations such as surface water and drinking water resources. Hence, the development of reliable and affordable technologies to remediate contaminated anoxic subsurface environments is needed to minimize the human health and environmental risks associated with 2,4-D.

**Fig. 1:**
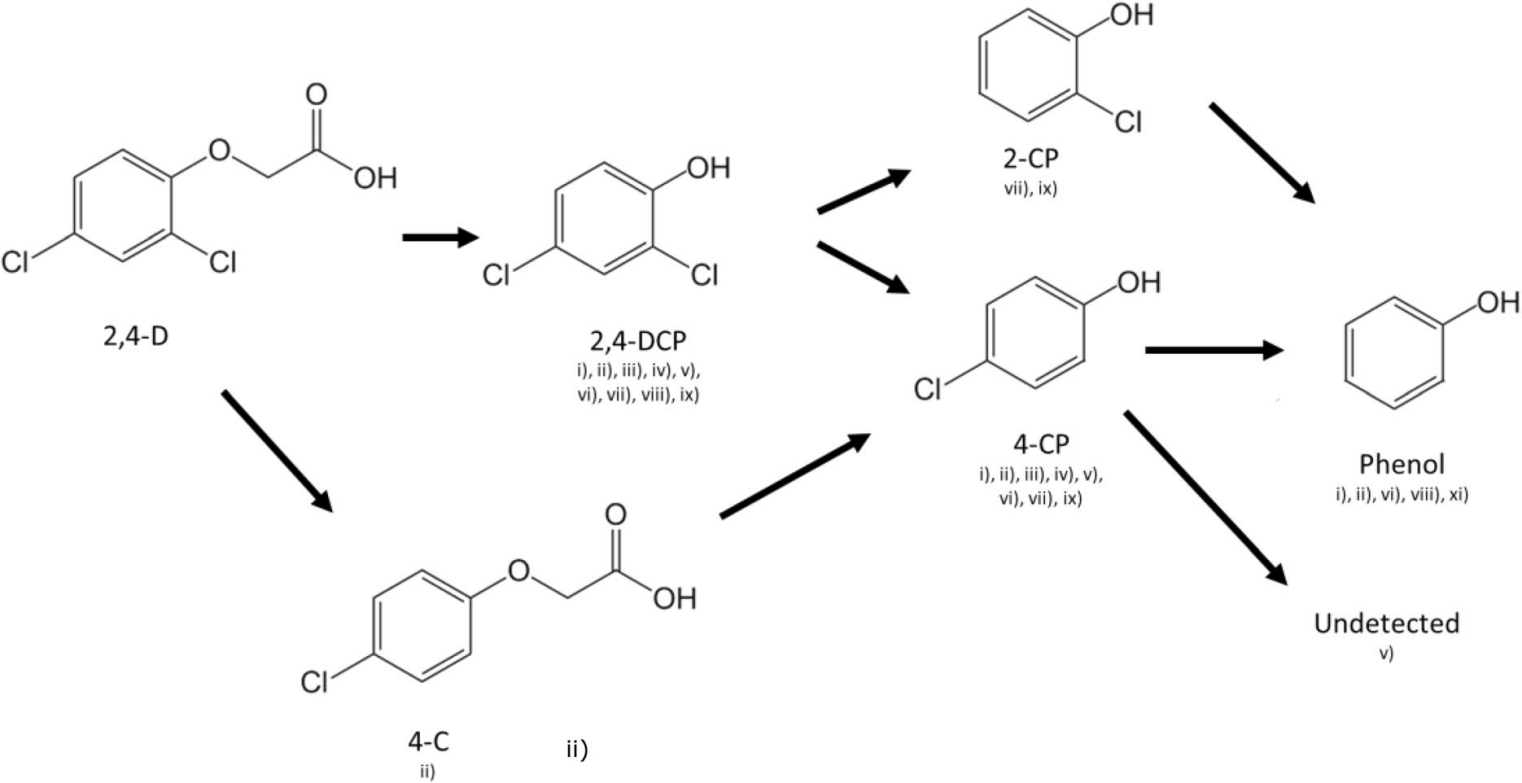
Biotransformation of 2,4-dichlorophenoxyacetic acid under anoxic conditions numbers below the compounds indicate the used references that detected those intermediates during the anaerobic microbial transformation of 2,4-D. (i) Gibson and Suflita (1986); (ii) Bryant et al. (1992); (iii) Boyle et al. (1999); (iv) Zipper et al. (1999); (v) Berestovskya et al. (2000); (vi) Roblez-Gonzalez et al. (2006); (vii) Wu et al. (2009); (viii) Yang et al. (2017). Abbreviations used: 2,4-D = 2,4-dichlorophenoxyacetic acid; 2,4-DCP = 2,4-dichlorophenol; 4-C = 4-chlorophenoxyacetic acid; 4-CP = 4-chlorophenol; 2-CP = 2-chlorophenol. Image produced on Chemsketch (ACD)

Bioremediation has been identified as a cost-effective and environmental friendly technology for the treatment of polluted environments (Azubuike et al. 2016). In addition, there are a growing number of reports on anaerobic microbial transformation of 2,4-D under methanogenic (Gibson and Sulfita 1986; Zipper et al. 1999; Berestovskya et al. 2000; Yang et al. 2017), sulfate-reducing (Boyle et al. 1999; Roblez-Gonzalez et al. 2006) and iron-reducing conditions (Wu et al. 2009). Different microbes have been associated with the removal of the acetic acid and chloride groups from 2,4-D (Boyle et al. 1999), such as bacteria belonging to the genera *Alcaligenes, Bulkoderia, Rhodoferax, Variovorax, Cupriavidus, Achromobacter, Comamonas, Holomonas* and *Pseudomonas* (Streber et al. 1987; Fulthorpe et al. 1995; Dom and Pamberton 1981; Zharikova et al. 2018). Although little is known about the enzymatic processes involved in anaerobic 2,4-D biodegradation, the identification of several metabolic intermediates has allowed inferring different degradation pathways (Fig. 1). The removal of chloride substitutes from 2,4-D and its metabolites were proposed to occur by reductive dechlorination, where 2,4-D acts as a terminal electron acceptor (Yang et al. 2017; Wang et al. 2009; Roblez-Gonzalez et al. 2006).

Reductive dechlorination can proceed through either co-metabolic processes or an energy-conserving metabolic mechanism known as organohalide respiration (OHR) (Smidt and de Vos 2004). The latter is mediated by organohalide-respiring bacteria (OHRB) that belong to distinct microbial groups in the phyla Chloroflexi, Firmicutes and Proteobacteria. Members of the genus *Desulfitobacterium* (Firmicutes) and proteobacterial genera *Geobacter* and *Sulfurospirillum* belong to the facultative OHRB with diverse metabolic capabilities with respect to the use of a variety of electron donors and acceptors including but not restricted to halogenated organic compounds (organohalides) (Futagami and Furukawa 2016). In contrast, members of *Dehalococcoides* (Chloroflexi) and *Dehalobacter* (Firmicutes) are obligate OHRB that only use organohalides as electron acceptors and hydrogen as an electron donor (Atashgahi et al. 2016). Therefore, 2,4-D and its chlorinated intermediates are likely reductively dechlorinated through OHR by OHRB. One such organism, the ferric iron-reducing *Comamonas koreensis* CY01, can directly reduce 2,4-D, although intermediates were not identified (Wang et al. 2009). Ha (2018) showed that *Thauera* sp. DKT could use 2,4-D and its intermediates 2,4-dichlorophenol (2,4-DCP), 4-chlorophenol (4-CP), 2-chlorophenol (2-CP) and phenol as a carbon and energy source under denitrifying conditions. Members of the genera *Dehalobacter* and *Desulfitobacterium* can respire, or are associated, with the reduction of 2,4-D and its intermediates 2,4-DCP, 4-CP and 2-CP (Wang et al. 2014; Li et al. 2013; Madsen and Licht et al. 1992; Thibodeau et al. 2004). Although co-metabolic reductive dechlorination of chlorinated aromatics was previously reported to be rare (Holliger et al. 2004), however recent research has also reported co-metabolic degradation of chlorinated compounds (Becker and Freedman 2014, Peng et al. 2020). *Dehalococcoides mccartyi* strain CBDB1 has been shown to co-metabolically dechlorinate 2,4-DCP to 4-CP (Adrian et al. 2007).

The aim of this investigation was to obtain enrichment cultures from Amazonian top (0–40 cm) and deep (50–80 cm) soil samples capable of 2,4-D transformation under methanogenic conditions. The influence of the soil sample depth and 2,4-D concentrations on the 2,4-D degradation capacity and the microbial community composition were monitored by metabolite analyses as well as Illumina MiSeq sequencing of PCR-amplified partial 16S rRNA genes and quantitative PCR (qPCR). This work contributes to further understanding of microbial 2,4-D degradation under methanogenic conditions and the involved microbial community, important insights for the development of pesticide bioremediation technologies.

## Materials and methods

### Soil sampling

Soil was sampled from Fazenda Nova Vida (10°10′58,11″ south, 62°49′20,74″ west), in Rondônia, Brazil. This farm has applied 2,4-D to control broadleaf weeds on its pasture for decades (Morais et al. 1996), and is a study location to compare physicochemical and microbiological aspects of the forest and pasture soil (Neill et al. 1995; Rodrigues et al. 2013). Soil samples were obtained at 0–40 cm (top soil) and 50–80 cm deep (deep soil) in August and October 2015, respectively, and were stored at 4 °C for 2 months until starting the microcosms.

### Microcosm setup

The 1st generation microcosms were set up with 60 g soil and 300 ml anaerobic medium (in 500 ml flasks with butyl rubber stoppers). The basal medium was prepared according to Zinder et al. (1984) with a N_2_:CO_2_ (70:30) atmosphere and amended with 10 ml L^−1^ of vitamin (Touzel and Albagnac 1983), buffered with 0.1% of NaHCO_3_ (100 g L^−1^) and reduced with 0.05% of Na_2_S.9H_2_O. Medium was supplemented with 1 mM acetate and 1 mM lactate as carbon source, and 5 µM 2,4-D as electron acceptor. The media was buffered at a pH of 7.0. Two sets of microcosms were prepared in triplicate, one with top soil, and another with deep soil. The microcosms were incubated in the dark at 30 °C, shaking at 150 rpm for 210 days with top soil material, and 90 days with deep soil material. During this period, 1 mM acetate and lactate were re-added at days 57 and 175 to top soil and at day 35 to deep soil microcosms.

**Fig. 2:**
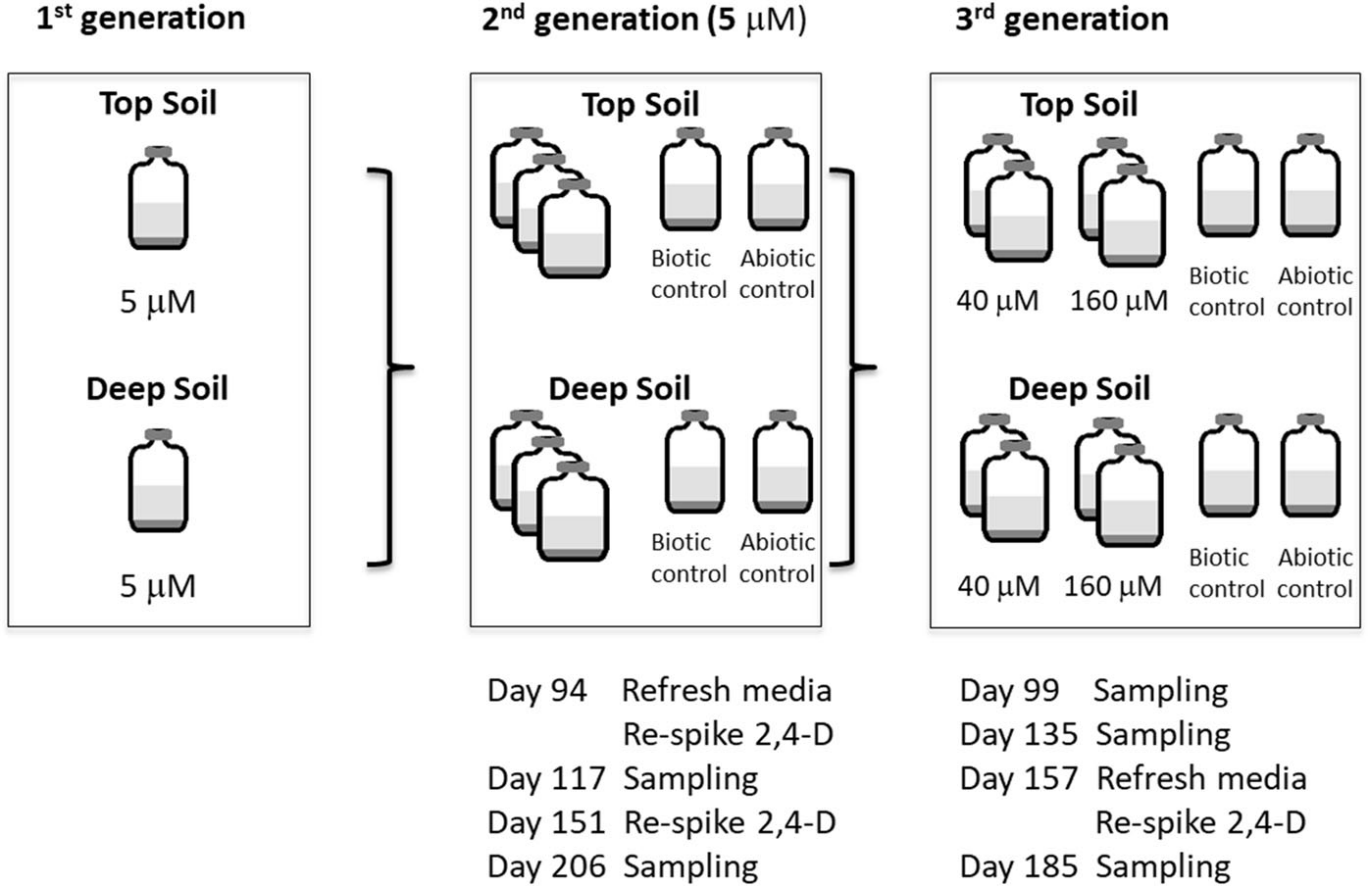
Schematic representation of the experimental set-up

These microcosms were used as inocula for the 2nd generation microcosms (2nd gen 5 µM microcosm), that were performed in triplicate. 40% inoculum (v/v) from the 1st generation microcosms was transferred into 120 ml bottles containing 50 ml of anoxic mineral medium prepared according to Holliger et al. (1998), amended with 10 mM acetate and 10 mM lactate, 5 µM 2,4-D, 5 µM 2-(4-Chloro-2 methylphenoxy) propionic acid (MCPP), and 5 µM 2,6-dichlorobenzamide (BAM) (Fig. 2). Abiotic controls were prepared similarly, with electron donors, pesticides and further addition of 2 mM NaN_3_ and 1 mM HgCl_2_. Biotic controls were also prepared similarly to biotic and abiotic microcosms, including electron donors, but without pesticides. The microcosms were incubated in the dark at 30 °C, and shaken at 150 rpm for 206 days. 10 mM acetate and 10 mM lactate were re-spiked on days 13, 39, 54, 67, 94, 110, 117, 125, 137, 151, 160, 171 and 177 to all bottles, except to the abiotic and biotic controls. Media was refreshed to prevent salt accumulation from the electron donor (potassium acetate and sodium lactate) by adding mineral medium (20 ml), pesticides (5 µM), acetate (10 mM) and lactate (10 mM) on day 94. Moreover, 5 µM of 2,4-D was supplied to the microcosms with top soil on days 94 and 151, and on day 94 to microcosms with deep soil, as 2,4-D depletion was observed in these bottles (Fig. 2).

To study the impact of higher concentrations of pesticides, 3rd generation microcosms (3rd gen) were prepared. 3rd gen 40 µM microcosms and 3rd gen 160 µM microcosms were prepared using 2nd gen microcosms sampled on day 117 as inocula (10% v/v). Conditions applied were identical to the 2nd gen microcosms, but two sets of higher concentrations of the pesticides were used, 40 µM and 160 µM for 2,4-D, MCPP, BAM and chloridazon, respectively. Abiotic controls and biotic control were prepared as above. Microcosms were incubated in the dark at 30 °C, shaking at 150 rpm for 185 days. Medium was refreshed and the cultures were re-spiked with 2,4-D to maintain the 2,4-D concentration on day 157, and acetate (10 mM) and lactate (10 mM) were re-spiked on days 21, 34, 47, 57, 63, 81, 85, 91, 99, 112, 125 and 137.

### Chemical analyses

Liquid samples were collected regularly from microcosms with pesticides and immediately centrifuged at 10,000 g for 10 min. Following centrifugation, the supernatant was transferred to an Eppendorf tube and stored at − 20 °C until analysis. The chlorinated pesticides 2,4-D, MCPP, BAM, chloridazon and the metabolic intermediates of 2,4-D transformation, 2,4-DCP, 2-CP, 4-CP, and phenol were analysed on a Dionex UltiMate 3000 RS’ HPLC–UV system (Thermo Fisher Scientific) installed with an Acquity UPLC CSH Phenyl-Hexyl 1.7 µm column (2.1 × 150 mm; Waters). Injections of 50 µL were performed by an auto-sampler, column temperature was set to 35 °C, and the flow was set to 0.3 mL min^−1^. To elute the pesticides, a binary gradient elution process was used with the following three stages: (1) 2 min with 75% solvent A (0.1% formic acid in water) and 25% solvent B (0.1% formic acid in acetonitrile); (2) 7 min with 45% solvent A and 55% solvent B; (3) 2 min with 75% solvent A and 25% solvent B. Transition between stages lasted 0.5 min. Data was analyzed with Xcalibur software (Thermo Fisher Scientific), and a detection limit of 0.02 mg/L was used for all compounds. Methane was measured with HP 5890 gas chromatography according to Steinbusch et al. (2008). First order 2,4-D degradation rate constants were calculated according to Yang et al. (2017).

### DNA extraction

Slurry samples (2 mL) were taken at day 117 and 206 from all 2nd gen microcosms, and at day 135 and 185 from all 3rd gen microcosms, and stored at − 20 °C until DNA extraction. DNA extraction was performed using a PowerSoil DNA isolation Kit (MO-BIO, USA) according to the manufacturer’s instruction. The quality and quantity of DNA was checked using a DS-11 FX Spectrophotometer/Fluorometer (DeNovix) and agarose gel electrophoresis.

### Quantitative PCR

16S rRNA gene copy number of *Dehalococcoides*, *Geobacter, Dehalobacter*, *Sulfurospirillum* and *Desulfitobacterium,* as well as total Archaea and total bacteria were quantitated using a C1000 Touch™ Thermal Cycler with a CFX384 Touch Real-Time PCR Detection System (Bio-Rad). The primers and qPCR program used are listed in Suplementary Table S1. Each reaction (final volume 10 μL) contained 5 μL iQTM SYBR Green supermix (Bio-Rad), 0.2 μL of 10 μM each primer, 2.6 μL nuclease free water (Promega) and 2 μL extracted DNA (diluted to 10 ng μL^−1^). Each reaction was run in triplicate and 3 non-template controls were included per experiment. The results were reported as the average of triplicate 16S rRNA gene copies per mL microcosms culture ± standard deviation. The quantification of *Dehalococcoides*, *Geobacter, Dehalobacter*, *Sulfurospirillum* and *Desulfitobacterium* on the last day of the experiment, were statistically compared among the different 2,4 -D concentrations (2nd gen 5 µM microcosms; 3rd gen 40 µM microcosms and 3rd gen 160 µM microcosms*,* and biotic control – without 2,4-D) and in different soil horizons by ANOVA and Test Tukey-Krument (Discroll 1996).

### Barcoded 16S rRNA amplicon synthesis and sequencing

A two-step PCR approach was used to generate barcoded amplicons of the V4 region of prokaryotic 16S rRNA genes. In the first step, universally tagged (Tian et al. 2016) UniTag1-515F forward primer (5′ GAGCCGTAAGCCAGTCTGC – GTGYCAGCMGCCGCGGTAA 3′) and UniTag2-806RB reverse primer (5′ GCCGTGACCGTGACATCG – GGACTACNVGGGTWTCTAAT 3′) were used (EMP, 2016). Each reaction (final volume 50 μL) contained 10 μL 5 × HF buffer (Thermo Fisher Scientific), 2.5 μL of forward and reverse primer (each 0.5 µM), 1 μL of 10 mM dNTPs (Promega), 0.5 μL of 2U μL^−1^ Phusion Hot Start II DNA polymerase (Thermo Fisher Scientific), 32.5 μL nuclease-free water (Promega) and 1 μL DNA, diluted to 10–20 ng μL^−1^. PCR was conducted using the following settings: 98 °C for 30 s followed by 25 cycles of 98 °C for 10 s, 50 °C for 20 s and 72 °C for 20 s with a final extension at 72 °C for 10 min. Five μL of PCR products were then electrophorized on a 1% (w/v) agarose gel (30 min at 100 V) containing 1 × SYBR Safe (Invitrogen) to confirm the fragment lengths (~ 300 bp). Primers targeting the universal tags were used in the second step to generate uniquely barcoded 16S rRNA amplicons. The barcodes can be found in the supplementary information (Table S2). Each reaction of the second PCR (final volume 100 μL) contained: 20 μL of 5 × HF buffer (Thermo Fisher Scientific), 5 μL barcoded forward and reverse primers (each 0.5 µM), 2 μL of 10 mM dNTPs, 1 μL of 2U μL^−1^ Phusion Hot Start II DNA polymerase (Thermo Fisher Scientific), 62 μL of nuclease-free water (Promega) and 5 μL PCR product from the first step PCR. PCR was conducted using the following settings: 98 °C for 30 s, followed by 5 cycles of 98 °C for 10 s, 52 °C for 20 s and 72 °C for 20 s with a final extension at 72 °C for 10 min. Five μL of PCR products were electrophorized on 1% (w/v) agarose gel to check fragment lengths. Second step PCR products were purified using the Highprep™ PCR kit (Magbio) according to manufacturer’s instructions, and the DNA concentrations were quantified using Qubit™ dsDNA BR Assay kit (Invitrogen) and a DS-11 FX Spectrophotometer/Fluorometer (DeNovix). Two hundred ng of DNA from each sample were pooled into a sequencing library, the resulting pool was purified and DNA concentrations quantified as before. The library was then sent to GATC Biotech (Germany) for Illumina MiSeq sequencing. Sequencing data were submitted to the European Bioinformatics Institute (EBI) under study accession number PRJEB37758.

### MiSeq sequencing data processing and analysis

Sequence analysis of the raw data was performed in NG-Tax 2.0 using default settings (Poncheewin et al. 2020). Amplicon sequence variant (ASV) picking was done for each sample; sequences were ordered by abundance and a sequence was considered valid when its cumulative abundance was ≥ 0.1%. Taxonomy was assigned using the SILVA reference database version 128 (Quast et al. 2013). Analyses of microbial communities were performed in R version 3.5 (R Core Team 2014). Beta-diversity (between sample diversity) was calculated using weighted and unweighted UniFrac with the phyloseq package (Mcmurdie and Holmes 2013).

## Results

### Biodegradation of 2,4-D

**Fig. 3:**
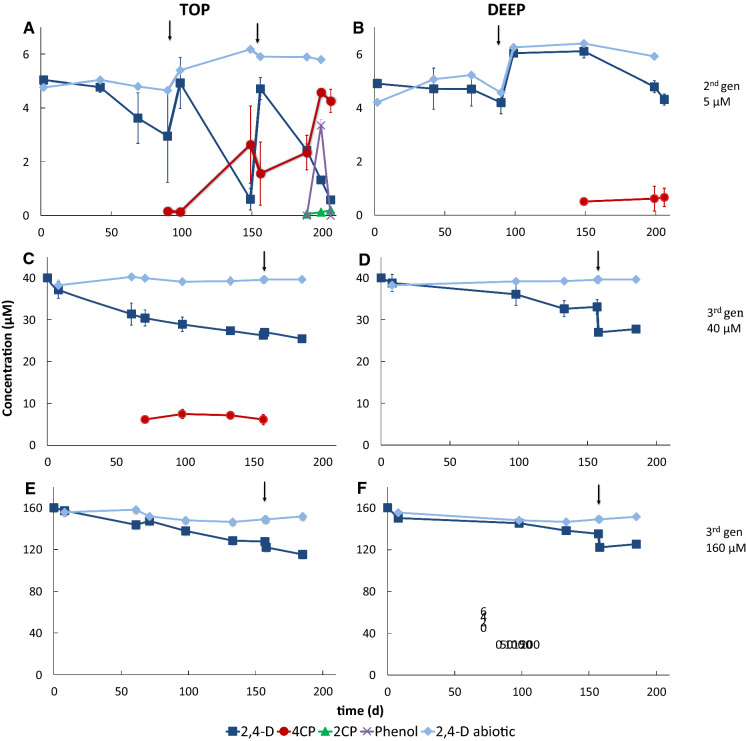
2,4-D and its transformation metabolites during the enrichment process. **a**, **b** cultures amended with 5 μM 2,4-D (2nd generation); **c**, **d** cultures amended with 40 μM 2,4-D (3rd generation); E–F: cultures amended with 160 μM 2,4-D (3rd generation). Error bars represent the standard deviation of analysis from replicate bottles (n = 3 for 5 µM cultures and n = 2 for 40 and 160 µM cultures). Arrows indicate when 2,4-D was re-spiked. *2,4-D*, 2,4-dichlorophenoxyacetic acid, *4-C* 4-chlorophenoxyacetic acid, *4-CP* 4-chlorophenol, *2-CP* 2-chlorophenol. Image produced on Chemsketch (ACD)

2,4-D transforming microbial enrichments under methanogenic conditions were developed with soil samples from the Amazon (Rondônia, Brazil). In general, 2,4-D depletion was observed in all microcosms, and top soil showed a faster depletion than deep soil (Fig. 3) Limited decrease of 2,4-D was observed in abiotic controls (Fig. 3). Methane production was observed in all microcosms throughout the experiment (Supplementary Figure S1), confirming that the observed 2,4-D transformation occurred under methanogenic conditions. Methane was not detected in abiotic controls.

The 2nd gen 5 µM microcosms with top soil were used to calculate transformation kinetics over a 206-day incubation period with 5 µM 2,4-D. The rate constant (k) of 2,4-D depletion increased from 0.006 d^−1^ following the first spike (day 0 to 90) to 0.042 d^−1^ following the second spike (day 99 to 149) and third spike (day 156 to 206). One of the degradation products, 4CP (4.26 µM), was measured at the end of the experiments in the microcosms containing top soil. Phenol (3.35 µM) was measured at day 199 and not detected anymore at the end of the incubation at day 206 (Fig. 3). The microcosms with deep soil showed a 2,4-D depletion rate constant of 0.017 d^−1^, with a limited decrease to 0.003 d^−1^ after the second spike at day 99. From day 99 to 206, 4CP was detected and reached a final concentration of 0.66 µM (Fig. 3).

The initial rate constant in the 3rd gen 40 µM top soil microcosms was 0.0027 d^−1^ (day 0 to 157) and decreased to 0.0021 d^−1^ (day 158–185) after the growth medium was refreshed. In the 3rd gen 160 µM top soil microcosms, the initial rate constant was 0.0014 d^−1^ (day 0 to 157), and 0.0021 d^−1^ after refreshing of the media. The microcosms with deep soil showed lower 2,4-D depletion rates of 0.0012 and 0.0010 d^−1^ in 3rd gen 40 µM and 3rd gen 160 µM microcosms, respectively (day 0 to 157).

Only the metabolite 4-CP was detected and quantified in the 3rd gen 40 µM microcosms. No additional metabolites were detected in all other 3rd gen 40 µM and 3rd gen 160 µM microcosms. 2,4-DCP was also detected in the 2nd gen 5 µM, 3rd gen 40 µM and 3rd gen 160 µM microcosms, but could not be quantified due to peaks overlapping with an unknown compound in the LC–MS analyses. Removal of MCPP, BAM or chloridazon was not observed in any of the 2nd or 3rd generation microcosms, and this data will not be discussed further.

### Abundance of total bacteria, archaea and OHRB

**Fig. 4:**
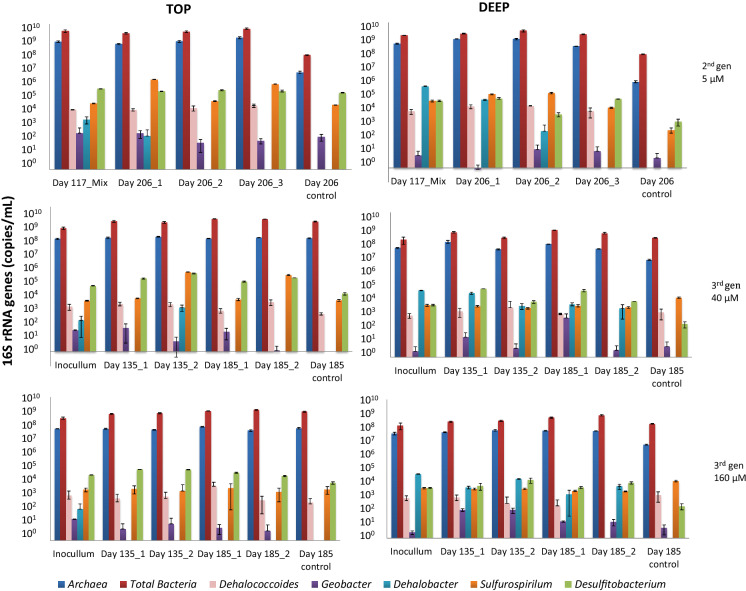
16S rRNA gene abundance of total bacteria, archaea and different OHRB during the microcosm experiments. Each qPCR value and error bar represents the average and standard deviation of triplicate reactions. 5 μM 2,4-D is data from 2nd generation, 40 and 160 μM are 3rd generation microcosms

The 16S rRNA gene copies of total bacteria and archaea per mL culture indicated that the community size remained relatively stable, within one order of magnitude in both top and deep soil microcosms (Fig. 4 and Supplementary Tables S2). The 16S rRNA gene copies of known OHRB such as *Dehalococcoides*, *Sulfurospirillum* and *Desulfitobacterium* were 10^4^–10^5^ copies/ml top soil culture in the last day of incubation in 5 µM 2,4-D. In higher concentration (40 and 160 µM 2,4-D), the 16S rRNA gene copies was 10^3^–10^4^/ml culture (Fig. 4 and Supplementary Table S2A). In deep soil enrichments, the 16S rRNA gene copies of *Dehalococcoides*, *Sulfurospirillum* and *Desulfitobacterium* were 10^4^ copies/ml culture in the last day of incubation in 5 µM 2,4-D and 10^3^–10^2^ copies/ml culture in 3rd gen (Suplementary S2B). *Dehalobacter* was only present at 10^3^ 16S rRNA gene copies/ml culture in the last day of incubation in deep soil (5 µM 2,4-D). *Geobacter* was not considerably present at the end of the incubations (concentration below 10^3^ gene copies mL^−1^). *Dehalococcoides* was not detected in the controls (without pesticides), whereas *Sulfurospirillum* and *Desulfitobacterium* were present in top soil enrichments at 10^5^ gene copies/ml microcosm.

Statistical analysis using ANOVA showed that only *Dehalococcoides* abundance was significantly different between the different microcosms (biotic control – without 2,4-D, 2nd gen 5 µM, 3rd gen 40 µM and 3rd gen 160 µM microcosms) with p value = 0.0004. The Tukey Test pointed out that this difference occurred between the sample of 2nd gen 5 µM microcosms and biotic control and between 2nd gen 5 µM and 3rd gen 160 µM microcosms with p value = 0.05 and 0.02, respectively.

### Microbial community dynamics

**Fig. 5:**
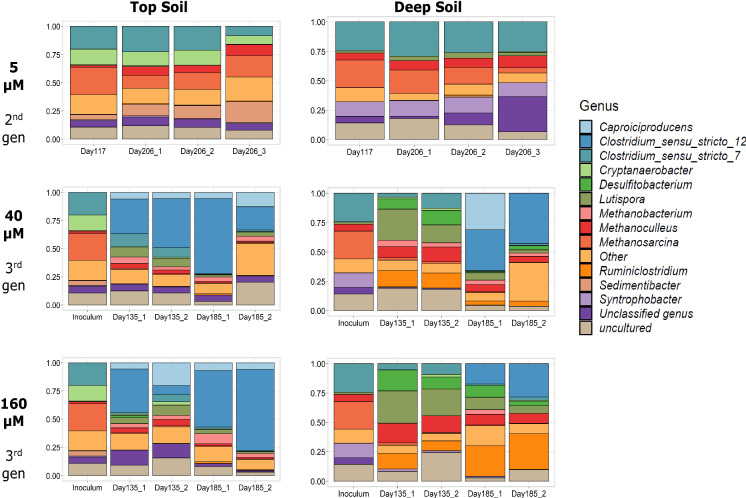
Relative abundance of the 15 most abundant genera in top and deep soil cultures at different 2,4-D concentrations. Other – represents the cumulative relative abundance of genera that is not part of the top 15. Inoculum for 3rd generation is the 5 µM microcosm from the 2nd generation, at day 117

In the 2nd gen microcosms (5 µM), the relative abundance of some groups changed with time for both top and deep soil microcosms, although with limited variation within the microbial composition (Fig. 5).

*Clostridium* was one of the most predominant groups among all the microcosms. *C.* sensu stricto 7 was predominant in the 2nd gen 5 µM microcosms, representing in terms of relative abundance up to 20% of the top soil and 24% of the deep soil microcosms on day 117. After 206 days, it represented around 8–20% of all sequences in the top soil and 26% in the deep soil. The relative abundance of *Clostridium* sensu stricto 12 in the 3rd gen 40 µM microcosms increased at day 135 for top soil, and at day 185 for deep soil. In the 3rd gen 160 µM top soil microcosms, the relative abundance of *Clostridium* sensu stricto 12 was 38% for one of the replicates at day 135, while the other replicate only showed around 7%. At day 185, between 50 to 70% of the microbial community belonged to that group. In the deep soil, for both 3rd gen 40 µM and 3rd gen 160 µM microcosms, the increase in relative abundance of *Clostridium* sensu stricto 12 became evident at day 185, where the relative abundance amounted to around 20% for both replicates.

**Fig. 6:**
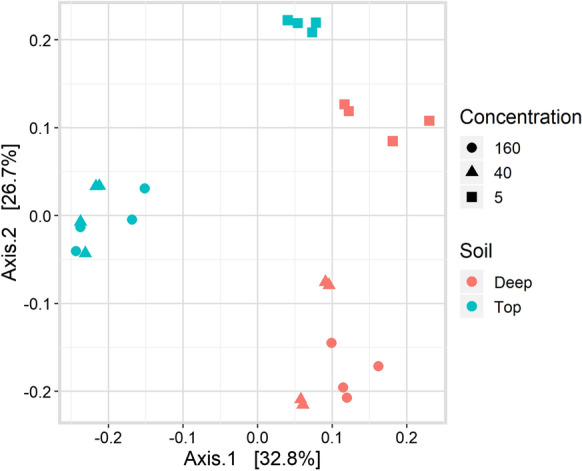
Principal coordinate analysis (PCoA) of microbial communities (ASVs) in top and deep soil based on unweighted unifrac distances

The microbial communities in the 2nd gen 5 µM microcosms for top and deep soil shared most of the predominant genera at day 117, and only differed by the presence of two microbial groups. In the top soil, *Cryptanaerobacter* and *Sedimentibacter* were present, while these two groups were not part of the most abundant genera in the deep soil. Furthermore, *Cryptanaerobacter* was no longer an abundant genus in the top soil 3rd gen 40 µM and 3rd gen 160 µM microcosms. The microbial composition from top and deep soil 2nd generation (5 µM) were more similar, in contrast to the 3rd gen 40 µM and 3rd gen 160 µM microcosms. Principal coordinate analysis (PCoA) revealed influence of 2,4-D concentration and soil origin on microbial communities (Fig. 6). The 2nd gen 5 µM microcosms*,* top and deep soil clustered with together, whereas the top 3rd gen 40 µM and 3rd gen 160 µM microcosms clusters were separated from the deep 3rd gen 40 µM and 3rd gen 160 µM microcosms (Fig. 6). Unfortunately, we cannot rule out the possibility that differences observed between 2nd gen 5 µM and 3rd gen 40 µM and 3rd gen 160 µM microcosms in terms of microbial composition are in part caused by the transfer, rather than the higher pesticide concentrations. Nevertheless, the lack of significant changes in microbial community over time during incubation of 2nd gen 5 µM microcosms that are in contrast to the significant changes in time in 3rd gen 40 µM and 3rd gen 160 µM microcosms*,* points towards a selective role of the pesticides.

## Discussion

### Degradation of 2,4-D

Our results show that 2,4-D can be degraded with material from an Amazonian agricultural soil that has received 2,4-D in the past to control broadleaf weeds on a pasture. The anaerobic biotransformation rate of 2,4-D was faster with top soil as inoculum than with deep soil, independent of the applied concentration of 2,4-D (Fig. 3). The majority of 2,4-D degradation studies focused on aerobic processes, with intermediates being detected and involved enzymes identified (Don and Pamberton 1981; Fukumore and Hausinger 1993; Furthorpe et al. 1995; Valey et al. 1996). Much less is known about anaerobic 2,4-D biotransformation and involved pathways. Some studies correlated 2,4-D anaerobic biotransformation with reductive dechlorination (Boyle et al. 1999; Wang et al. 2009), whereas others added carbon sources such as acetate, glucose, propionate, butyrate, and sucrose (Boyle et al.^1999^; Roubles-Gonzales et al. 2006; Wang et al. 2009; Yang et al. 2018; Luo et al. 2019). In our study, we used Brazilian soil as inoculum to study 2,4-D biotransformation. We found only one other study, related to Amazon samples and 2.4-D. However, this group studied the resistance of aerobic microorganisms to 905 µM of 2,4-D, but not its degradation (Mussy et al. 1999).

The 2,4-D depletion rates decreased in our 3rd gen 40 µM and 3rd gen 160 µM 3rd microcosms. Two factors can be associated with this. Possibly, crucial growth compounds in the soil were reduced upon transferring from one generation to the other, and during refreshing of the medium, resulting in a reduced degradation rate. Wang and He (2013) have reported the importance of sediments or sediment substitutes during the enrichment of polychlorinated biphenyl dechlorinating cultures. In addition, soils are a source of humic acids, that have been shown to facilitate the reductive dechlorination of 2,4-D (Wang et al. 2009). A second possible explanation is toxicity effects of 2,4-D at higher concentrations. Organohalogens are known to inhibit methanogenesis at higher concentrations. While Wu et al. (2009) demonstrated that a pure culture of *C. koreensis* CY01 was capable of degrading 2,4-D at concentrations up to 180 µM, 20 µM greater than the concentrations used here, that study did not fully explore toxicity effects on transformation rates. As our results seem to suggest that toxicity of 2,4-D could affect transformation, we suggest future work to identify 2,4-D toxicity thresholds.

In the top soil microcosms, 2-CP and phenol were detected, which are known intermediates of 2,4-D reductive dechlorination (Gibson and Suflita, 1986; Bryant et al*.* 1992; Roblez-Gonzalez et al*.* 2006; Yang et al. 2017) (Fig. 3). Moreover, 4-CP was produced but not degraded further in 2nd gen 5 µM and 3rd gen 40 µM microcosms, indicating 4-CP as a terminal product of 2,4-D biotransformation that was also observed in some of the deep soil cultures, over the time frame monitored during this study. Other studies have similarly reported 4-CP as a final product of 2,4-D biotransformation (Boyle et al. 1999; Zipper et al. 1999; Roblez-Gonzalez et al. 2006). In 3rd gen 160 µM microcosms, only 2,4-DCP was detected (not quantified), suggesting an incomplete biotransformation of 2,4-D, possibly due to a toxic effect of the higher 2,4-D concentration.

### Characterization of microbial communities involved in 2,4-D biodegradation

Microbial community composition seems to be affected by both the origin of the inoculum and the generations with different 2,4-D concentration (Fig. 6). For all the tested 2,4-D concentrations, top soil microbial communities transformed 2,4-D faster than deep soil communities. Regarding the different generation microcosms, the 2,4-D depletion rates were higher at 2nd gen 5 µM microcosms than at higher 2,4-D concentrations (3rd gen). In top soils at 2nd gen 5 µM microcosms, 2,4-D, we found two microbial groups that could be associated with 2,4-D biodegradation. One of these groups is *Cryptanaerobacter,* a microbial group with members capable of transforming phenol, a degradation product of 2,4-D, into benzoate (Juteau et al. 2005). We did note phenol depletion in some of our microcosms, but as we did not measure benzoate, we cannot confirm a similar phenol transformation. The second group that was present in top soil is *Sedimentibacter,* previously shown to support the growth of *Dehalobacter* in soil enrichments fed with acetate or lactate (Van Doesburg et al. 2005). *Sedimentibacter* was previously involved in the dehalogenation of β-hexachlorocyclohexane (β-HCH), which is a organochlorine micropollutant, similar to 2,4-D (Van Doesburg et al. 2005). Metagenome analysis showed that *Sedimentibacter* in co-culture with *Dehalobacter* synthesized cobalamin (vitamin B12), an essential cofactor for reductive dehalogenases (Maphosa et al. 2012). We hypothesize that the contribution of *Sedimentibacter* in our top soil microcosms could be also cobalamin production.

In our study, qPCR assays showed no enrichment of *Dehalococcoides, Sulfurospirillum* or *Desulfitobacterium* during 2nd and 3rd generation assay. Moreover, *Dehalobacter* and *Geobacter* strongly decreased in numbers, indicating that they were most likely not involved in 2,4-D transformation (Fig. 4 and Table S2 Supplementary). The presence of OHRB indicates that these microorganisms were present in the Amazon soil. However, our results indicate that these microorganisms were not further enriched during transfer from 2nd gen to 3rd gen microcosms.

The transformation of 2,4-D in our microcosms could likely be due to co-metabolism. Co-metabolic reductive dechlorination mediated by vitamin B12 has been demonstrated for other chlorinated compounds such as chloroform, tetrachloromethane, tetrachloroethene, among others (Gantzer and Wackett 1991, Workman et al. 1997, Smidt and de Vos 2004, Guereiro-Barajas and Field 2005; Becker and Freedman 2014, Peng et al. 2020). The genus *Clostridium* was one of the most dominant groups among all microcosms (Fig. 5), and *Clostridium* that has been identified before as one of the main genera involved in cometabolic transformation of 2,4-D (Yang et al. 2018). Furthermore, members of the genus *Clostridium* are also known for their potential for co-metabolic transformation of chlorinated compounds using enzymes involved in the Wood-Ljungdahl pathway (WLP) (Peng et al. 2020). Unfortunately, we did not monitor cobalamin, but as mentioned before, we observed 2,4-D transformation, and no enrichment of OHRB. This fact, combined with the presence of vitamin B12/cobalamin producing microorganisms in high abundance, could be evidence of that cometabolic dechlorination contributes or even dominates 2,4-D biotransformation observed in this work.

## Conclusions

To the best of our knowledge, this study is the first report of microbial communities in Brazilian soil samples involved in anaerobic 2,4-D transformation, with samples from the Amazon forest. 2,4-D transformation was observed and its depletion rate decreased slightly in the 3rd generation, indicating no enrichment occurred from the 2nd to the 3rd generation. This could have been caused by the increase in concentration of 2,4-D from 5 µM to 40 or 160 µM, and/or that simply more time was needed in the 3rd generation incubations to activity to be re-established. Chlorinated phenols and phenol were detected as intermediates.

qPCR showed no notable enrichment of OHRB *Dehalococcoides, Sulfurospirillum* and *Desulfitobacterium*. 16S rRNA gene sequencing indicated that the genus *Clostridium* was one of the most dominant groups among all microcosms that are known for their potential for co-metabolic organochlorine transformation. Differences in microbial community composition between the various microcosms could be explained by the origin of the inoculum and the 2,4-D concentration.

To conclude, the top soil microcosms showed a better 2,4-D degradation than the deep soil microcosms. Our results are a first step towards understanding the fate of 2,4-D in Amazon soil and contribute to the development of bioremediation strategies in South American forests.

## Supplementary Information

Below is the link to the electronic supplementary material.

Supplementary file1 (DOCX 123 kb)
