# Supplementary material for: 2,4-Dichlorophenoxyacetic acid degradation in methanogenic mixed cultures obtained from Brazilian Amazonian soil samples
Source: Biodegradation. 2021 Apr 20;32(4):419–33. doi: 10.1007/s10532-021-09940-3 (PMC8260542; doi:10.1007/s10532-021-09940-3)
Supplement: Supplementary file 1 — Supplementary file1 (DOCX 123 kb) [file 10532_2021_9940_MOESM1_ESM.docx]

Table S1. Primers used for qPCR in this study

| Target | Name ^a^ | Oligonucleotide sequence (5´–3´) | Reference for primer | Reference for qPCR program |
| --- | --- | --- | --- | --- |
| Total bacteria | Eub341F | CCTACGGGAGGCAGCAG | (Muyzer et al 1993) | (Atashgahi et al 2013) |
|  | Eub534R | ATTACCGCGGCTGCTGGC |  |  |
| Archaea | ARC787F | ATTAGATACCCSBGTAGTCC | (Yu et al 2005) | (Yu et al 2005) |
|  | ARC1059R | GCCATGCACCWCCTCT |  |  |
| *Desulfitobacterium* | Dsb406F | GTACGACGAAGGCCTTCGGGT | (Smits et al 2004) | (Smits et al 2004) |
|  | Dsb619R | CCCAGGGTTGAGCCCTAGGT |  |  |
| *D. mccartyi* | Dco728F | AAGGCGGTTTTCTAGGTTGTCAC | (Smits et al 2004) | (Atashgahi et al 2013) |
|  | Dco944R | CTTCATGCATGTCAAAT |  |  |
| *Dehalobacter* | Dre441F | GTTAGGGAAGAACGGCATCTGT | (Smits et al 2004) | (Atashgahi et al 2013) |
|  | Dre645R | CCTCTCCTGTCCTCAAGCCATA |  |  |
| *Geobacter* | Geo196F | GAATATGCTCCTGATTC | (Amos et al 2007) | (Azizian et al 2010) |
|  | Geo535R | TAAATCCGAACAACGCTT |  |  |
| *Sulfurospirillum* | Sulfuro114F | GCTAACCTGCCCTTTAGTGG | (Duhamel et al 2006) | (Sutton et al 2015) |
|  | Sulfuro421R | GTTTACACACCGAAATGCGT |  |  |

^a^ Primer names may not correspond to original publication

Table S2. qPCR quantification of 16S rRNA gene copy numbers of Total *Bacteria*, *Archaea*, *Geobacter*, *Dehalobacter*, *Dehalococcoides*, *Sulfurospirilum*, *Dessulfitobacterium* in Top Soil Enrichment 2^nd^ Generation (5 μM/L) and 3^rd^ Generation (40 and 160 μM/L). Each qPCR value represent the average value obtained from triplicate reactions of triplicate assay


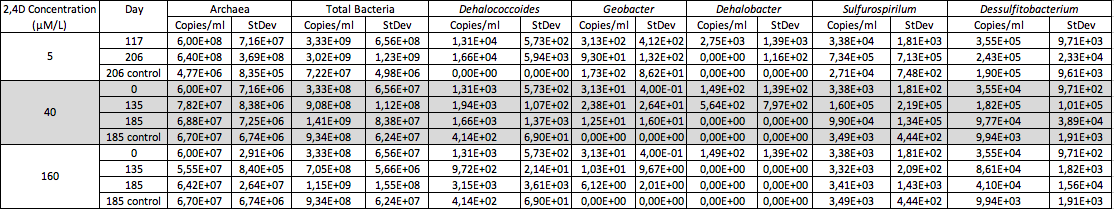


StDev – Standard Deviation

Table S3. qPCR quantification of 16S rRNA gene copy numbers of Total *Bacteria*, *Archaea*, *Geobacter*, *Dehalobacter*, *Dehalococcoides*, *Sulfurospirilum*, *Dessulfitobacterium* in Deep Soil Enrichment 2^nd^ (5 μM/L) and 3^rd^ Generation (40 and 160 μM/L). Each qPCR value represent the average value obtained from triplicate reactions of triplicate assay.


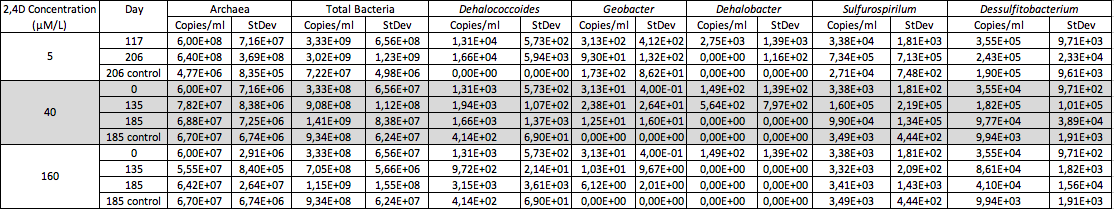


StDev – Standard Deviation


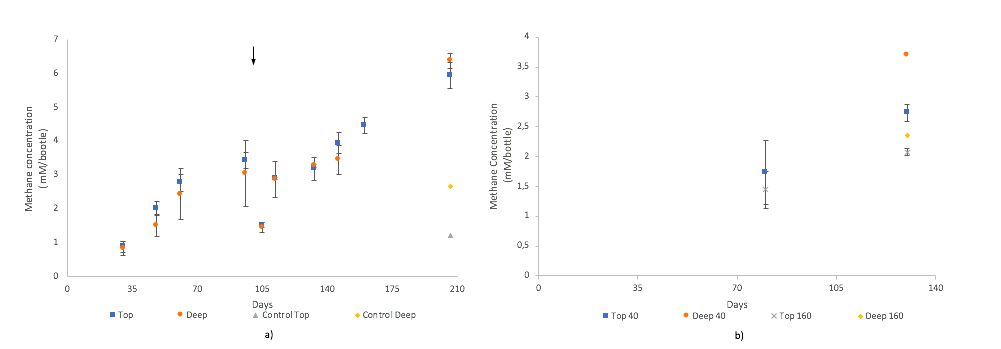


Legend: The arrow indicate when the medium was refreshed.

Figure S1. Methane production in 2nd Generation microcosms 5 M 2,4-D (a) and 3rd generation 40 µM and 3rd generation 160 µM (b).
